# The use of locking plates in proximal humeral fractures: Comparison of outcome by patient age and fracture pattern

**DOI:** 10.4103/0973-6042.63214

**Published:** 2009

**Authors:** Michael Leonard, Leibo Mokotedi, Uthman Alao, Aaron Glynn, Mark Dolan, Pat Fleming

**Affiliations:** Department of Trauma and Orthopedic Surgery, Cork University Hospital, Cork, Ireland

**Keywords:** Locking plates, proximal humeral fractures, PHILOS

## Abstract

**Purpose:**

This study was undertaken to evaluate the efficacy of a proximal humeral locking plate, and to specifically study the effect of patient age and fracture type on the outcome.

**Materials and Methods:**

Thirty-one cases of proximal humeral fractures fixed by using the proximal humeral interlocking (PHILOS) plate were reviewed.

**Results:**

Average functional scores (minimum 18 months post operation) per AO / ASIF fracture type were 25.3 for type A, 21.4 for type B, and 22.7 for type C. There was no statistically significant difference between the groups. The functional scores for patients over 65 years of age were significantly inferior (*P* = 0.03). At a final radiological review (mean 12 months post operation), 30 (96%) of the patients demonstrated fracture union. Seven patients (22.5%) required a second surgical procedure.

**Conclusion:**

We obtained both good functional results and bone healing with the PHILOS plate, irrespective of fracture type; the older patients had a poorer outcome. We caution the surgeons on the high potential for reoperations with its use.

## INTRODUCTION

Proximal humeral fractures are common, accounting for 5 to 9% of all fractures.[1] Their incidence is particularly high in patients over 65 years of age, in whom they represent one of the most common fracture types.[2,3] Most proximal humeral fractures are stable, minimally displaced, and can be managed conservatively.[4]

The surgical treatment of displaced unstable fractures, however, remains a challenge. Non-operative management of these more severe fractures is associated with poor results.[4] The large range of operative techniques described (e.g., K wiring, tension band wiring, plating, nailing, arthroplasty) for managing the more complex fractures is a testament to the lack of clear superiority of any one method.[5–9] Most of these techniques have been associated with complications related to hardware failure, osteonecrosis, non-union, malunion, rotator cuff impairment, and impingement.[10]

Proximal humeral locking plates, such as the proximal humeral interlocking (PHILOS) plate, (Synthes, Switzerland) offer several potential advantages in the treatment of these injuries. They are site-specific, low-profile plates. The plate is precontoured for the proximal humerus, and the insertion of locking screws obviates the need for a plate-to-bone compression, preserving the blood supply to the bones. The insertion of multiple polyaxial locking screws through the specific targeting device into the humeral head fragment provides a fixed-angle support in multiple planes, which should, in theory, maintain the reduction achieved,[1] while allowing for early mobilization. However, in spite of all the potential benefits, significant levels of construct failure and revision surgery with the use of proximal humeral locking plates have been reported, particularly in patients over 65 years of age.[11]

This study was undertaken to evaluate the use of the PHILOS plate system for the treatment of proximal humeral fractures. We specifically wanted to examine the effectiveness of the PHILOS plate on different fracture patterns, the impact of patient age and of the humeral neck-shaft angle attained following fixation, on the outcome.

## MATERIALS AND METHODS

From May 2003 to May 2007, 31 consecutive patients with displaced fractures of the proximal humerus had open-reduction and internal fixation with a PHILOS plate (Synthes, Switzerland). The patients were identified from the trauma database of a single university based level 1 trauma center.

There were 23 women and nine men with a mean age of 61.6 years (19 to 86). Twenty- five of the patients sustained their injury following a fall, five from a road traffic accident, and one from direct assault.

Fractures were classified with the AO/ASIF system;[12] there were eight type A (extra-articular unifocal), 15 type B (extra-articular bifocal), and eight type C (articular) fractures. All fractures met the indications for operative treatment outlined by Neer *et al*.,[13] that is, an angulation of the articular surface of more than 45 degrees or displacement between the major fracture fragments of more than 1 cm. It is our policy to treat some fracture-dislocations (particularly in the physiologically elderly), head-splitting fractures, and impression fractures that involve over 40% of the articular surface, with hemiarthroplasty.

Using the immediate anteroposterior postoperative radiograph the humeral neck-shaft angle was determined. The normal anatomic neck-shaft angle of the humerus is considered to be approximately 130°.[14,15]

The radiographic follow-up consisted of plain radiographs on the second postoperative day, at six weeks, and every three months after that, for approximately one year.

The mean time for union was 12 weeks (9 to 20).

### Operative technique

All cases were performed by a senior orthopedic surgeon. The patients received prophylactic intravenous antibiotics. All patients were placed in the beach-chair position and the C-arm was positioned parallel to the patient at the head of the bed. Satisfactory imaging was ensured before prepping the patient. A delto-pectoral approach was used, with minimal soft tissue dissection. The biceps tendon was identified and retracted, and the fracture exposed. On occasion the biceps tendon was found to be interposed in the fracture fragments requiring mobilization. Traction sutures were then placed around the tendon-bone interfaces of the rotator cuff and the tuberosity fragments. The head fragment, when involved, was then reduced from its typical varus position through manipulation and flexing of the arm. Once in position the traction sutures were used to bring the fragments beneath the head to buttress the articular fragment. The facture was then held temporarily with K wires and the reduction checked fluoroscopically. The traction sutures were then passed through the proximal eyelets on the plate without any tension. The PHILOS plate was then applied lateral to the bicipital groove, 1 – 2 cm distal to the upper end of the greater tuberosity. A conventional non-locking screw was then inserted into the slotted gliding hole on the plate, which brought the plate to the bone and allowed for minor adjustments in the plate height and position when checked on fluoroscopy. The proximal targeting device was then used to insert the polyaxial locking screws into the head; locking screws were also inserted into the shaft. In one patient with poor bone stock Allomatrix bone substitute (Wright Medical, USA) was used. The traction sutures were then tied down to the plate and final images taken.

The arm was placed in a sling after wound closure. Only pendular exercises were permitted for the first four weeks postoperatively, with elbow and wrist range of motion also encouraged. Passive progressing to active range of motion was then commenced under the guidance of a physiotherapist, 4 – 6 weeks postoperatively. Resistive strengthening was begun when fracture union was ensured.

The postoperative outcome was measured with the Quick Disabilities of the Arm, Shoulder, and Hand Outcome Measure (QuickDASH), at a minimum of 18 months postoperatively (range 18 – 60 months). The QuickDASH is an eleven-item questionnaire that has been validated for either proximal or distal disorders of the upper limb.[16] The total score ranges from 0 to 100 points, with 100 indicating the highest disability. The functional outcome using DASH has been rated as excellent (< 20 points), good (20 – 39 points), fair (40 – 60 points) or poor > 60 points.

Radiologic outcome measurements recorded at a mean of 12 months (range, 10 – 15 months) postoperatively included, bone-union, defined as the continuity of cortex visible on at least two radiographic views, avascular necrosis, loss of fixation and / or hardware failure.

### Statistical analysis

Statistical analyses were performed with the SPSS 13 (SPSS, Chicago, Illinois). Comparisons were made using the Mann-Whitney *U* tests. A *P*-value of less than 0.05 was considered to be significant.

## RESULTS

The mean operative time was 81 minutes (range, 60 – 123) and the mean blood loss was 222 ml (range, 150 – 600). Two patients developed superficial wound infections, and both responded to intravenous antibiotics. No neurovascular injuries occurred. The average clinical follow-up period was 14 months (range, 12 – 18).

Twenty seven patients (87%) responded to the DASH questionnaire. Postoperative Quick DASH scores ranged from 0 to 93.2 (mean = 22.7). The four patients who did not respond had undergone an uneventful recovery, had united their fractures radiologically, and had been discharged from the clinic.

Average DASH scores per AO / ASIF fracture type were 25.3 for type A, 21.4 for type B, and 22.7 for type C. There was no statistically significant difference between these groups.

The mean DASH score for patients under 65 years of age (n = 14) was 21.5, and it was 27.5 for patients over 65 years of age (n = 13). The difference was statistically significant (*P* = 0.03).

There was a trend for patients with intraoperative restoration of the humeral head–neck angle to greater than 90 degrees (n = 15, mean angle 126°) to have a better outcome (mean DASH score = 20.4) than those who were fixed with an angle of under 90 degrees (n = 12, mean angle 84°, mean DASH score 24.3). However, this was not statistically significant.

At the final radiologic review (mean 12 months post operation) 30 of the patients had their fractures united clinically and radiologically (96%) [Figures 1a and b].

**Figure 1a:**
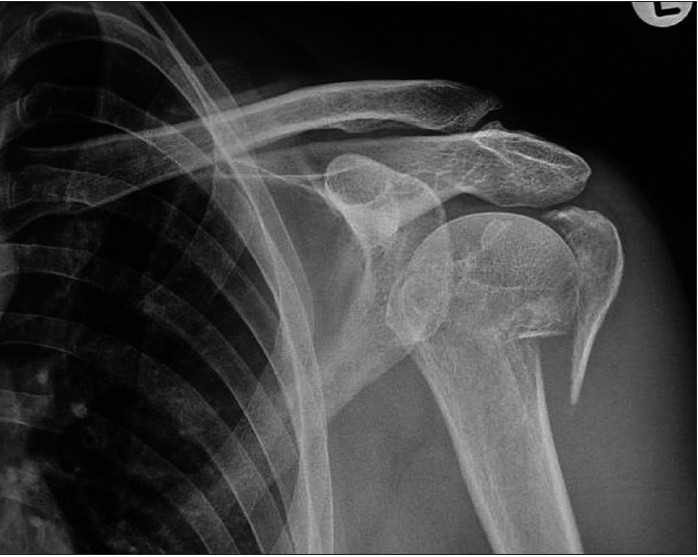
Preoperative anteroposterior radiograph of a 54-year-old female with a four-part fracture in her left proximal humerus

**Figure 1b:**
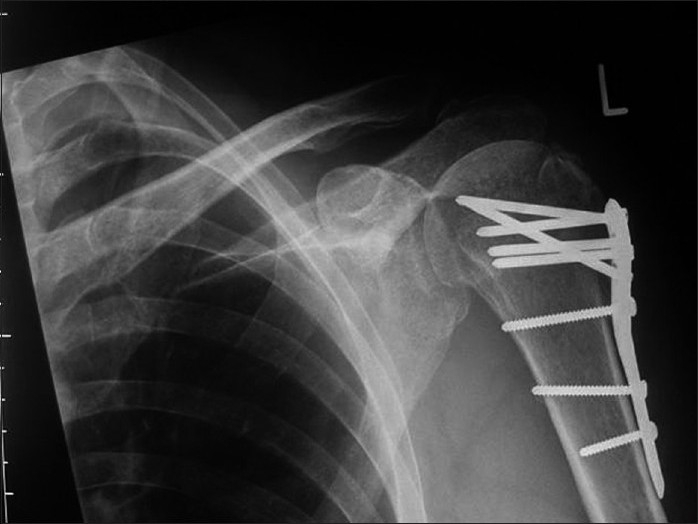
Note the multiple angled screw fixation and solid bony union evident on the postoperative radiograph at eight months, with no signs of avascular necrosis

All complications were diagnosed within 12 months of the initial surgery. Seven patients (22.5%) required a second surgical procedure. Three patients failed to have their fractures united after initial fixation; one a 19-year-old female had only autologous bone grafting performed [Figures 2a and b], the other two (both over 65 years of age) underwent plate removal, bone grafting, and intramedullary nailing. Both the young female patient and one of the patients over 65 years of age eventually united at their fracture sites, but the other patient did not and subsequently had a hemiarthroplasty performed seven months after PHILOS plate fixation. Two patients required removal of the plate, which in both cases had been placed in an excessively superior position causing symptomatic impingement. One patient required removal of a prominent screw and one patient required a manipulation under anesthesia for a frozen shoulder following fracture healing. Avascular necrosis (AVN) was observed in two patients, both of whom had AO / ASIF type C fractures. In both cases only a small percentage of the humeral head was involved; the fracture healed and there was no perforation of the humeral head by any of the screws.

**Figure 2a:**
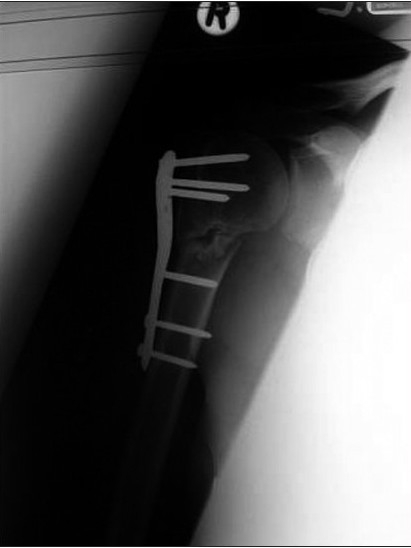
A completely displaced proximal humeral fracture in a 19-year-old-girl. The fracture was treated with a PHILOS plate, but had not united four months postoperativley

**Figure 2b:**
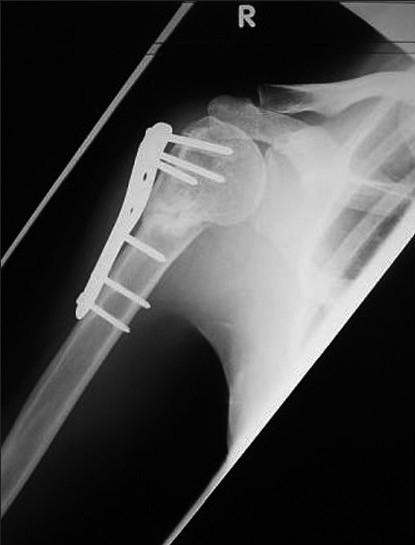
Autogenous iliac crest cancellous graft was subsequently inserted and the fracture united six months postoperativley

## DISCUSSION

Proximal humeral fractures are challenging to treat. Despite being common injuries there are no clear-cut indications for any of the various surgical options described.[4] Defining correct treatment guidelines through analysis of current treatment options is becoming increasingly important, as the prevalence of osteoporotic fractures of the proximal humerus are expected to rise in the next three decades, and the functional outcome achieved after treatment may determine a patient's level of independence.[17]

The PHILOS plate was designed to improve screw fixation and minimize soft tissue dissection. It attempts to achieve these aims through a combination of multidirectional locking screws for the head, precontouring of the plate, and locking screws in the shaft[18]. The clinical results to date have been mixed.[1,11,18]

This study evaluated the clinical and radiological results of the PHILOS plate used in 31 patients, over a four-year period in a University based Level 1 trauma center.

There are a number of limitations to our study; patient numbers' were relatively low (31) and there was a significant loss of follow up (4 / 31 = 13%). The outcome assessment was only subjective, with no objective clinical examination of range of movement or function. There was also a wide variation in patient age, mechanism of injury, and fracture type. In the radiographic analysis we evaluated the bony union of tuberosities, but we did not define the position of union which may affect rotator cuff function, and hence the outcome.

We found no significant difference in the functional outcome using DASH scoring, after PHILOS plate fixation between fracture types, using the AO / ASIF classification system, for a minimum of 18 months postoperatively. We could find only one other article that compared fracture type before PHILOS fixation with a clinical outcome. Bjorkenheim *et al*., in their study reported a reducing trend in the clinical outcome related to the severity of the fracture at a minimum follow-up of one year, but they did not report any statistical analysis of their results.[19] However, the three cases of non-union and the two cases of AVN that we report have all occurred in the more severe fracture types B and C.

The impact of age on the outcome after PHILOS plate fixation is of interest, particularly as there is a general belief that these plates provide a more secure fixation in the osteoporotic bone.[19] We found a significantly inferior clinical outcome in patients over 65 years of age. Moonot *et al*., demonstrated no significant difference in the functional outcome between the under and over 65 year olds at a mean follow up of 11 months post PHILOS plate fixation.[18] We recorded two cases of non-union and one case of AVN in the over 65 group. The inferior functional outcome and complications in the elderly population is probably multifactorial, combining both lower strength and reduced range of motion, with a more tenous blood supply and healing capacity. We encountered no mechanical failure of the plate and screws. The use of local adjuvants, such as, bone graft or bone graft substitutes at the time of surgery, particularly when poor bone stock is encountered, may well improve the rate of union and has been advocated by others.[4,18]

There was statistically no significant difference in the clinical outcome between those who had restoration of their humeral head–neck angle to greater than 90 degrees at the time of surgery and those who did not. As with all locking plates, fracture reduction must be achieved prior to plate application, this can be technically demanding. We achieved this in only 17 of our cases (54%). It has been shown that unstable proximal humeral fractures have a tendency toward varus collapse, even in the presence of locking plate fixation. This can lead to varus deformity with impingement and potential screw cut-out. While we have not encountered this problem to date, we advocate optimal restoration of the head-neck angle to guard against this potential complication.

The fact that seven (22.5%) of our patients required a second procedure following PHILOS plate fixation is a cause of concern. Three of these reoperations were a result of technical error. In one case a screw was left too long, and in the other two cases the plate was placed in an excessively superior position causing symptomatic impingement. Previous authors have described a high incidence of screw perforation with the use of locking plates, for proximal humeral fractures.[20,21] Based on these reports we were conscious at all times to limit screw length as much as possible while still obtaining a secure fix in the fracture fragments. The overall reported rate of complications following PHILOS plate fixation is high, Owlesy *et al*., reported a radiographic complication rate in 36% of their patients, with a 43% rate of cut-out in patients over 60 years of age.[5] Moonot *et al.* reported significant complications in 21% of their cases.[10] Of the three patients in this study who developed a symptomatic non-union, two were over 65 years of age and had sustained a complex fracture type. A hemiarthroplasty in this situation is an option, the possible benefits of which include; a single operation, excellent pain relief, reasonably good function, and no potential for non-union or AVN.[3] However, the results obtained in the recent studies of hemiarthroplasties for trauma have been mixed.[22,23] Problems with strength, function, range of motion, neurological deficits, reoperations, and displacement of both the prosthetic head and tuberosities have all been reported.[22]

Although the number of patients in our study was relatively small and it was not a randomized controlled study, the results demonstrate both the potential benefits and problems of using the PHILOS plate. We obtained good functional results and bone healing in a vast majority of our patients. There was no statistical difference in the functional outcome between the fracture types at a minimum of 18 months postoperatively. Patients under 65 years of age had a significantly better outcome. The PHILOS plate is a useful addition to the armamentarium of the trauma surgeon, however, we caution all surgeons on the high potential for complications and reoperations with its use.
